# Low-Level Laser Therapy in the Treatment of Recurrent Aphthous Ulcers: A Systematic Review

**DOI:** 10.1155/2015/150412

**Published:** 2015-03-23

**Authors:** Fernando Alves Vale, Maria Stella Moreira, Fernanda Campos Souza de Almeida, Karen Muller Ramalho

**Affiliations:** ^1^School of Dentistry, Ibirapuera University (UNIb), 04661-100 São Paulo, SP, Brazil; ^2^Department of Social Dentistry, School of Dentistry, University of São Paulo (USP), 05508-000 São Paulo, SP, Brazil

## Abstract

Recurrent aphthous ulcers (RAUs) are the most common lesion found in the oral cavity. There is no definitive cure for RAUs and current treatments are aimed at minimizing symptoms. Since low-level laser therapy (LLLT) modulates inflammatory responses, and promotes pain reduction and cellular biostimulation, LLLT can be suggested as an alternative treatment for RAUs. The literature concerning the potential of LLLT in the treatment of RAUs was evaluated. A systematic literature review identified 22 publications, of which only 2 studies were adopted. The eligibility criteria consisted of randomized controlled trials (RCTs). Both RCTs achieved significant results concerning LLLT and pain-level reductions and reduced healing times. Despite the variance in irradiation conditions applied in both studies, very similar wavelengths were adopted. There is accordingly strong evidence that wavelength plays an important role in RAU treatment. Taking into account the different parameters applied by selected RCTs, it is not possible to suggest that a specific protocol should be used. However, in light of the significant results found in both studies, LLLT can be suggested as an alternative for RAU treatment. Additional RCTs should be performed in order to reach a clinical protocol and better understand the application of LLLT in RAU treatment.

## 1. Introduction

Recurrent aphthous ulcers (RAUs), also known as canker sores or aphthae, are frequent lesions that affect the oral cavity [1]. These ulcerations affect 5–66% of the population [2]. The lesions are characterized by recurrent bouts of single or multiple rounded, flat, painful oral ulcers [2]. These ulcers result in oral epithelium wounds, which have exposed nerve endings and are associated with pain [3]. The pain inhibits patients' abilities to eat, drink, and maintain oral hygiene [4]. RAUs typically appear with grey-white pseudomembranes surrounded by thin erythematous halos [2]. These lesions typically occur in the nonkeratinized mobile oral mucosa [5, 6]. The normal progression of the lesions requires 10–14 days for healing.

Three clinical subtypes of RAUs have been established according to magnitude, number, and duration of outbreaks: minor, major, and herpetiform [7, 8]. The minor RAUs represent 70–85% of all cases and manifest themselves as small, rounded, or oval lesions, covered by a grayish-white pseudomembrane and surrounded by an erythematous halo [7, 8]. Minor RAUs episodes typically involve the appearance of 1–5 ulcers measuring less than 1 cm in diameter. These episodes are self-limiting and heal within 4–14 days without leaving scars [9–11]. Major RAUs are the most severe form of the disease and represent 10% of all cases [8–10, 12]. Major RAUs measure over 1 cm in size and tend to appear on the lips, soft palate, and pharynx. The lesions persist for over 6 weeks and can leave scars [8–10, 12]. The herpetiform subtype accounts for 1–10% of all cases and is characterized by recurrent outbreaks of small, deep, and painful ulcers. Several aphthous ulcers, measuring 2-3 mm in size, can develop simultaneously, even though they can merge and form larger ulcerations [8–10, 12].

The etiology of RAUs is not clear [13]; however a series of factors are known to predispose an individual to the disease. The primary predisposal factors include genetic factors, alpha-hemolytic streptococcal infections [14, 15], a decreased immune system integrity, and deficiencies in folic acid, iron [15, 16], or vitamin B12 [15, 17]. Additional predisposing factors include stress, trauma [15, 16, 18, 19], allergies to certain foods [15, 16, 18], and endocrine imbalances [15, 16, 18, 19]. Immune alterations have been observed, beginning with an unknown antigenic stimulation of the keratinocytes and resulting in the activation of T lymphocytes, cytokine secretion, including tumor necrosis factor-alpha (TNF-*α*), and leukocyte chemotaxis. TNF-*α* is believed to play an important role in the development of RAUs. TNF-*α* has been found to be increased 2–5-fold in the saliva of affected patients [20]. Alterations in salivary enzyme defense system have been reported in affected patients [21]. An increase in lymphocyte infiltration of the epithelium is reported, which prompts ulcer formation [8–10]. On the other hand, many systemic diseases are known to be associated with RAUs, including Chron's disease, ulcerative colitis [22], Behcet's syndrome [23–25], hematological disorders, vitamin deficiencies, gastrointestinal diseases, cyclic neutropenia, Reiter syndrome, periodic fever, aphthous pharyngitis and cervical adenopathy, and immune deficiencies [26, 27].

The diagnosis of RAUs is based on patient anamnesis and clinical symptoms. There is no specific diagnostic test for RAU, though it is essential to discard possible underlying systemic causes. It is prudent to request a complete series of laboratory tests, including a complete blood count, and evaluation of iron, vitamin B12, and folic acid. A biopsy of the lesion is only recommended in the case of diagnostic uncertainty, since the findings only indicate a simple nonspecific inflammatory lesion [8–10].

Since the main etiology of RAS is still unknown, a definitive cure does not exist and the present treatments are aimed at alleviating the symptoms [28]. Some treatments have been suggested; however, such treatments are palliative, not curative [28]. Current treatment options include topical analgesic and anesthetic agents, corticosteroids, antibiotics, multivitamins, cauterization, and a variety of combined therapies [1]. Most of the treatments are associated with side effects or other disadvantages that make their usage clinically questionable [28]. A challenge to patient management is to significantly stimulate the healing process and minimize patient discomfort, without side effects [29].

Low-level laser therapy (LLLT) is also known as “soft laser therapy,” “laser phototherapy” (LPT), and “cold laser therapy.” Since LLLT modulates inflammatory responses with a reduction in oedema and pain and cellular biostimulation, this therapy could be considered to be an alternative treatment for RAUs [47]. This systematic literature review aimed to assess studies of LLLT used for the treatment of RAUs in terms of pain reduction and wound healing. An analysis of the quality of the studies was performed in order to gather state of art evidence about the use of LLLT for the treatment of RAUs.

## 2. Materials and Methods

A systematic review of the relevant literature was conducted via database research. Literature searches were conducted independently by two authors using the following databases: in the following databases until 1 June, 2014, MEDLINE, Pubmed, Embase, Cochrane Database, LILACS, and Google Scholar. Two authors independently extracted the data in duplicate. Mesh terms were used individually or combined in appropriate language forms: “aphthous,” “laser therapy,” “low-level laser therapy, and LLLT.” Twenty-two articles were identified (Table 1). Articles in languages other than English, German, Portuguese, and Spanish were excluded;* in vitro* studies, as well as cases reports, were also excluded. Eligibility criteria consisted of randomized controlled trials (RCTs). Considering these criteria, 2 eligible studies were isolated.

## 3. Results and Discussion

Out of the 22 publications found in databases concerning LLLT in RAU treatment (Table 1), only two eligible studies were selected [1, 4].

Both eligible RCT studies applied LLLT for the treatment of minor RAUs [1, 4]. Albrektson et al. (2014) [4] developed a randomized, single-blinded, placebo-controlled clinical trial with predetermined inclusion and exclusion criteria. Forty patients with RAUs participated in the study (*n* = 20/group). The laser parameters described by the authors were GaAlAs semiconductor laser with a wavelength of 809 nm, 60 mW, 1800 Hz, a duration of 80 seconds per treatment, and a dose of 6.3 J/cm^2^ [4]. In the treatment group, the laser tip was in direct contact with the ulcer, for a duration of 80 seconds; in the placebo group, the same procedure was conducted, but without any power [4]. LLLT was applied on three occasions, each separated by 24 hours. In the study of Albrektson et al. (2014) [4], patients were asked to rate their pain on a visual analogue scale (VAS) and also discuss their subjective experience of eating, drinking, and brushing their teeth before the placebo or laser treatment and also on subsequent days [4]. In the laser group, the pain score significantly decreased on day 1 (VAS rating: 84.7 to 56.2) and on day 2 (VAS rating: 56.2 to 31.5) (*P* < 0.0001) [4]. In the placebo group, the pain score changed from 81.7 to 80.7 (days 0 to 1) and to 76.1 on day 2 [4]. All participants at the start of the trial had difficulty eating that was either moderate or severe. In the laser group, 75% of the participants had moderate or severe difficulty eating on day 1 [4]. Twenty percent of the participants had moderate or severe difficulty eating on day 2, and none of the patients had difficulty eating on day 3 [4]. In the placebo group, all participants had moderate or severe difficulty eating on days 1 and 2 [4]. On day 3, 85% of patients still had moderate or severe eating difficulty (*P* < 0.0001). The same results were obtained with drinking liquids. On day 0, 90% of patients in the laser group and 80% of the patients in the placebo group reported moderate or severe difficulties drinking [4]. On day 1, 50% of the participants in the laser group had moderate or severe difficulties drinking (*P* < 0.001) [4]. On day 2, only 5% of participants in the laser group had moderate or severe difficulties drinking; in the placebo group, 80% of patients had difficulties drinking on both days 1 and 2 (*P* < 0.001) [4]. The difference was not as stark for participants who reported severe difficulty brushing their teeth. On day 1, 50% of the patients in the laser group reported severe difficulty brushing their teeth, compared with 75% of patients in the placebo group (*P* < 0.006). On day 2, only 5% of patients in the laser group had severe difficulty brushing their teeth, compared with comparison 65% of patients in the placebo group (*P* < 0.0001) [4]. The study of Albrektson et al. (2014) revealed that LLLT promoted a highly significant analgesic effect in acute minor aphthous ulcers in comparison with the placebo group and also significantly reduced pain levels associated with eating, drinking, and brushing teeth [4].

In the second selected study, Aggarwal et al.  (2014) [1] developed a sham-controlled, split-mouth study with predetermined exclusion and inclusion criteria. Thirty patients with two minor RAUs in their oral cavity participated in the study [1]. The study assessed pain reduction, lesion size, and healing time. In each patient, one of the ulcers was randomly allocated to be treated with LLLT [1]. The laser parameters described by the authors were as follows [1]: diode laser, 810 nm, and 0.5 W [1]. The treatment consisted of one appointment with four sequential sessions of LLLT applications, each lasting 45 seconds with a gap of about 30–60 seconds between each session, for a total laser application time of about 3 minutes [1]. The application of the laser was done in noncontact mode, with a distance of 2-3 mm between the laser tip and the surface of the ulcer [1]. The laser beam was applied in a continuous circular motion, covering the entire ulcer surface. For the ulcers included in the sham group, the same technique was done without activating the laser unit [1]. Immediately after LLLT application, complete pain relief was observed in 28 of the 30 patients in the LLLT group [1]. The LLLT group showed a statistically significant reduction in pain compared with the sham control group (*P* < 0.001) [1]. The complete resolution of the ulcers in the active group required 3.05 ± 1.10 days, compared with 8.90 ± 0.45 days in the sham control group. Compared with the sham group, authors found the complete healing time for the LLLT group to be highly significant, with a *P* value less than 0.001 [1].

Although both selected studies [1, 4] were the most well designed studies found in the literature concerning LLLT in the treatment of RAUs, both manuscripts still omit certain critical pieces of information. Both studies do not discuss the use of a power meter to check the power output before laser irradiation. It is not uncommon for laser equipment to have true power outputs that deviate from the stated power levels. Studies should always measure the power output before laser irradiation. Additionally, the method of randomization used in both studies was not described.

Both studies used different ranges of power in their treatment of minor RAUs (60 mW [4] and 0.5 W [1]). Furthermore, the studies differed in other respects as well: contact [4] and noncontact [1] of the laser tip with tissue and three irradiations in one day [1] versus one irradiation per day for three days [4]. The studies nonetheless obtained highly significant treatment results. Therefore, despite the variance in irradiation conditions, the authors used very similar wavelengths in their treatments: 809 nm [4] and 810 nm [1]. In their meta-analysis, Enwemeka et al. (2004) [48] studied the effect of low-power lasers (<500 mW) on conditions such as sores and ulcer wounds and concluded that laser therapy is effective at repairing tissue and controlling pain, although the outcomes may be influenced by the wavelength of the laser. Some studies concerning acute pain also revealed that lasers operating at infrared wavelengths led to more effective pain reduction [49–52]. Other studies in the literature that were not selected for this review once were not controlled clinical trials used other laser wavelengths, including 633 nm [29], 670 nm [41], and 904 nm [38]. These studies did not find significant differences in their results. There is accordingly strong evidence that wavelength plays an important role in the final results of RAU treatment. Future studies are encouraged to test the influence of different wavelengths on pain control and reducing the sizes of RAUs.

Clinical and laboratory evidence lends support to the use of low-level lasers to promote wound repair [53–57], as well as reduce pain [48, 58] and inflammation [59–64]. Increased blood flow and capillary vasodilatation were observed after LLLT, which are known to promote healing [65]. These effects include lymphocyte stimulation, activation of mast cells, and increased ATP production. Furthermore, the proliferation of various types of cells such as fibroblasts [63], macrophages, epithelial [66], and stem cells [67] was observed. All of these combined factors promote anti-inflammatory and biostimulatory effects, thus enhancing wound healing [56, 68, 69]. The activation of mast cells leads to the release of proinflammatory cytokines, which promotes local leukocyte infiltration of tissues. Since mast cells play a key role in leukocyte functions, the modulation of mast cell activity by LLLT can be of considerable importance in promoting wound healing in oral cavities [65]. Increased proliferation, maturation, and locomotion of fibroblasts have been noted as side effects of LLLT. In addition, a reduced production of prostaglandin E2 (PGE2) and an increase in the production of basic fibroblast growth factor have been observed [70, 71]. These effects may improve wound healing.

One mechanism related to pain relief that has been proposed is the modulation of pain perception by modification of nerve conduction via the release of endorphins and enkephalins [47, 72]. An additional mechanism described is related to enhanced ATP synthesis in the mitochondria of the neurons [73]. When ATP synthesis is reduced, the consequence is mild depolarization, which decreases the threshold of triggering an action potential. On the contrary, an increase in ATP synthesis, which is caused by LLLT, will induce hyperpolarization and obstruction of stimuli, which would thus reduce the induction of pain stimuli [73]. The mechanism of increased ATP synthesis after LLLT is dependent upon absorption by photoreceptors in mitochondrial components, specifically in the electron transport (respiratory) chain [74]. In addition, the inhibition of prostaglandin E2 and interleukin-1 beta also help in reducing pain (PG increases pain by sensitizing the receptors by lowering their thresholds) [75]. Moreover, described study found that conduction of nerve fibers was inhibited by LLLT, due to a reversible conformational change in the voltage-gated Na-K channels [76].

Immune mechanisms appear to play an essential role in the pathogenesis of RAUs and likely involve a cell-mediated immunoresponse mechanism, with the generation of T cells and TNF-*α* by leucocytes (macrophages and mast cells) [77]. LLLT could affect the inflammatory process, causing a diminution in some cytokine levels, such as interleukin-1b (IL-1b), TNF-*α*, and interferon-g (IFN) [78]; however, their effects on RAUs should be investigated further.

Taking into account the fact that there are few eligible RCTs published in the literature concerning LLLT for the treatment of minor RAUs, it is not possible to dictate that a specific protocol should be used by clinicians. However, in light of the two significant results found in both studies, LLLT can be thought of as an encouraging alternative for RAU treatment, without side effects. Since the effect of LLLT in the treatment of other clinical subtypes of RAUs (major and herpetiform) was not assessed in the selected studies, future RCTs studies are encouraged to evaluate this effect. RCTs with larger samples and multicenter studies should also be developed in order to enrich our knowledge about the application of LLLT in all RAUs subtypes. Furthermore, long follow-ups of patients were not performed by the two selected RCTs. It is important to observe the frequency of the lesions before and after LLLT treatment. Follow-up analysis is strongly recommended.

## 4. Conclusions

Considering that there are just two controlled clinical trials published in the literature concerning LLLT for the treatment of minor RAUs, it is not possible to dictate that a specific protocol should be used. However, in light of the two highly significant results found in both studies, LLLT can be suggested to be a positive alternative for the treatment of RAUs. Even so, additional studies should be performed in order to support and establish a clinical protocol for all RAUs subtypes, as well as elucidate the specific mechanisms by which LLLT can promote the pain relief from and healing of RAUs.

## Figures and Tables

**Table 1:** Publications found in databases up until June 1, 2014.

Authors	Year
Howell et al. [29]	1988
von Ahlften [30]	1987
Mikhaǐlova et al. [31]	1992
Parkins et al. [32]	1994
Contreras et al. [33]	1994
Neiburger [34]	1995
Acuña Castro and Ovalle Castro [35]	1997
Prikuls [36]	2000
Bladowski et al. [13]	2004
Eman and Hussein [37]	2002
Kashmoola et al. [38]	2005
Monteiro and Tonani [39]	2007
Khademi et al. [40]	2009
de Souza et al. [41]	2010
van As [42]	2011
Caputo et al. [3]	2012
da Silva Marciano et al. [43]	2012
Anand et al. [44]	2013
Rozo et al. [45]	2013
Misra et al. [46]	2013
Aggarwal et al. [1]	2014
Albrektson et al. [4]	2014
